# *Helicobacter pylori *vacuolating toxin A and apoptosis

**DOI:** 10.1186/1478-811X-9-26

**Published:** 2011-11-01

**Authors:** Joachim Rassow

**Affiliations:** 1 Ruhr-Universität Bochum, Institut für Physiologische Chemie, Medizinische Fakultät, Gebäude MA3, D-44780 Bochum, Germany

**Keywords:** *Helicobacter pylori*, vacuolating cytotoxin A, apoptosis, lipid rafts, endosomes, vacuoles, mitochondrial targeting, mitochondrial inner membrane, ion channel, cytochrome c

## Abstract

VacA, the vacuolating cytotoxin A of *Helicobacter pylori*, induces apoptosis in epithelial cells of the gastic mucosa and in leukocytes. VacA is released by the bacteria as a protein of 88 kDa. At the outer surface of host cells, it binds to the sphingomyelin of lipid rafts. At least partially, binding to the cells is facilitated by different receptor proteins. VacA is internalized by a clathrin-independent mechanism and initially accumulates in GPI-anchored proteins-enriched early endosomal compartments. Together with early endosomes, VacA is distributed inside the cells. Most of the VacA is eventually contained in the membranes of vacuoles. VacA assembles in hexameric oligomers forming an anion channel of low conductivity with a preference for chloride ions. In parallel, a significant fraction of VacA can be transferred from endosomes to mitochondria in a process involving direct endosome-mitochondria juxtaposition. Inside the mitochondria, VacA accumulates in the mitochondrial inner membrane, probably forming similar chloride channels as observed in the vacuoles. Import into mitochondria is mediated by the hydrophobic N-terminus of VacA. Apoptosis is triggered by loss of the mitochondrial membrane potential, recruitment of Bax and Bak, and release of cytochrome c.

## Review

VacA, the vacuolating cytotoxin A, is one of the major virulence factors released by *Helicobacter pylori*. VacA is a protein of about 88 kDa that easily assembles in defined oligomeric complexes, forming anion channels in target membranes. The name refers to the capability of the toxin to cause a formation of large vacuoles in cultured cells [1,2]. However, VacA is also implicated in other activities, including interactions with the immune system, modifications of the permeability of polarized epithelial cell monolayers, and induction of apoptosis [3,4]. The first observations of VacA-dependent apoptosis were published more than 10 years ago [5,6]. Since then, numerous projects on VacA were carried out, but it was difficult to reconcile the divergent results. Only recently, a unifying model is emerging [7,8], suggesting that VacA-induced cell death is essentially dependent on a peculiar traffic route of VacA inside the host cells.

In this review, we will first ask for the evidence that VacA is of any relevance for the induction of cell death in the infected tissues. The next section gives a short summary on the structure and the molecular properties of the toxin. The following sections give a detailed description of the traffic route of VacA: I, binding to target cells, II, lipid rafts and endocytosis, III, vacuolation, IV, mitochondria. In the final section, we will discuss the possible relation between mitochondrial targeting and VacA acting as a trigger of apoptosis.

## The cellular effect: VacA triggers apoptosis

*H. pylori *has a unique capability to survive in the mucus layer of the stomach. In many cases, the infection is acquired early in childhood and persists throughout the entire life of the host [3,8]. About 80-90% of the bacteria are mobile within the mucus, a fraction of 10-20% is found in direct contact with the surface of the epithelial cells [9]. Some bacteria may even enter host cells and survive for some time in an intracellular compartment [8,10]. Under normal physiological conditions, the epithelial cells of the gastric mucosa are constantly replaced by new cells with a 3-5 day renewal rate. The cells undergo apoptosis, the remnants are exfoliating into the gastric lumen [11,12]. The rate of this process is significantly increased upon infection by *H. pylori *[13-15]. Remarkably, even excessive apoptosis of the epithelial cells is not necessarily a reason for ulcer formation. In most cases, the integrity of the epithelium is maintained by the induction of a secondary hyperproliferative response. The mucosal cells are replaced by cells migrating from the neck segments of the gastric glands [16]. In fact, about half of the world population is infected by *H. pylori*, but only in about 15% of the cases the infection causes peptic ulceration [17-19].

What is the reason for the increased rate of cell death in the presence of *H. pylori*? Initial studies concentrated on a possible involvement of the CD95 (APO-1/FAS) system [20-22]. It was observed that infection with *H. pylori *entailed an enhanced Fas receptor expression. However, the reason of this effect was not clear. Moreover, data on other pathogens demonstrated that bacteria are able to trigger apoptosis by many different means, including specific effector proteins that act inside the host cells [23]. VacA was already known to be one of the major virulence factors of *H. pylori*, and it had been observed that VacA is able to enter different target cells. It was therefore tested if this protein may be sufficient to trigger apoptosis. In fact, several independent studies confirmed that cells incubated with purified VacA showed all signs of apoptosis [5,6,24-26]. It is now generally assumed that several effects contribute to apoptosis in *H. pylori*-infected tissues, including a contribution of the extrinsic, receptor-mediated pathway [27-29]. However, it is clear that *H. pylori*-induced apoptosis is also possible independently of death receptors [5,6,24-26,30], and the VacA toxin appears to be the most prominent mediator [4,31].

VacA-dependent effects are also observed with leukocytes and it is well possible that this activity is of much higher pathological significance as compared to the enhanced turnover of the epithelial cells.

Infection of the mucosa by *H. pylori *is accompanied with a significant disruption of the epithelial barrier function, permitting access of VacA to the gastric submucosa [3,32,33]. Already very low concentrations of VacA are sufficient to causes an efficient inhibition of the proliferation of the T cells due to down-regulation of Interleukin-2 (IL-2) transcription [34-37]. Moreover, the number of T cells can also be reduced independently of IL-2, possibly by apoptotic depletion [38-40]. Similar as with epithelial cells, apoptosis of T cells was originally thought to be due to death receptor signalling but later shown to be mediated by the mitochondrial pathway [30]. Apoptosis was reported both for T- and B-cell lines [41]. Moreover, *H. pylori *is also able to cause apoptosis of human monocytes and macrophages. Again, cell death appears to depend on a participation of mitochondrial factors [42,43]. There is some indication that all subsets of leukocytes, including granulocytes and dendritic cells, may be susceptible to VacA [36]. However, there are also some data suggesting that in particular granulocytes and dendritic cells may be resistant against VacA-dependent apoptosis [42,44].

Although there is compelling evidence that *H. pylori *virulence factors are able to trigger apoptosis of different leukocytes if applied *in vitro*, data are lacking to estimate the relevance of leukocyte apoptosis and the role of VacA in the infected tissues. It was questioned if the density of *H. pylori *in the mucus layer is sufficient to achieve a sufficient concentration of VacA to trigger apoptosis of leukocytes in the submucosa [33]. However, the bacteria are obviously successful in establishing a state of drastic immune suppression in their immediate vicinity. It may be speculated that inhibition of IL-2 activities and induction of apoptosis correspond to two different lines of defence which allow the bacteria to survive in the immediate neighbourhood of a chronic inflammation.

## The structure: VacA forms hexameric anion channels

What are the features that enable VacA to trigger cell death? VacA is initially synthesized in the bacteria as a single polypeptide of about 140 kDa, in most cases comprising 1.287 residues [45-48]. The protein is subsequently released by type V-secretion. In this context, the N-terminal leader sequence of 33 residues and the C-terminal autotransporter domain of about 33 kDa are cleaved, yielding a mature toxin of 88.2 kDa [49]. The polypeptide can be further processed into an N-terminal fragment of 33.4 kDa (named p34 or p37, residues 1-311) and a C-terminal fragment of 54.8 kDa (named p55 or p58, comprising residues 320-821), both parts stay associated by non-covalent interactions. The relevance of the cleavage is not clear, it is not required for VacA activity. However, many data have shown that the N-terminal domain is essential in the toxic activity of VacA while the C-terminal domain is essential in binding to target membranes [3,4].

VacA is able to assemble in flower-shaped hexameric structures. These can form both free in solution and after insertion in membranes. The complexes can have a mass of nearly 1000 kDa, indicating that they may contain 12 VacA polypeptides [50-52]. The complexes probably form by association of two donut-like hexamers. They dissociate into monomers at pH < 4.5 [51]. At least under some conditions, VacA also forms stable heptameric complexes, however, hexamers represent the dominant form of the toxin [52].

Probably the most important feature of the VacA toxin is its capability to form anion channels of low conductivity [53-55]. Oligomeric VacA can act as a chloride channel of about 10 pS. The conductivity is completely inhibited by the nonspecific chloride channel blocker NPPB (5-nitro-2-(3-phenylpropylamino)benzoic acid [55,56]. N-terminal segments of p58 may be involved in pore formation [57], however, we recently found that isolated p34 alone is sufficient to form the ion channel [58]. Unfortunately, so far no crystal structure of the holo-toxin was resolved. The N-terminal part of VacA is prone to aggregation, making it difficult to obtain crystals. The structure of the ion-conducting channel is therefore unknown. The C-terminal parts of VacA were crystallized separately some years ago, the structure revealed an impressive array of β-sheets [59]. Eventually, it should be noted that not only the precise structure of VacA is unknown, but also the topology of the VacA complexes in membranes. The determination of the VacA structure and membrane topology is still one of the big challenges in the field of VacA research.

## The traffic route: (I) Binding of VacA to target cells

Electrophysiological studies showed a successful integration of functional VacA into artificial membranes in the absence of any accessory proteins. However, targeting to host cells is obviously facilitated by distinct receptor structures [60]:

### Epithelial cells

Association of VacA with epithelial cells was found to involve interactions with RPTPα and RPTPβ, two structurally unrelated receptor-like protein tyrosine phosphatases [61-65]. The relevance of VacA binding to these phosphatases was questioned because it is unclear if binding of VacA to RPTPα or RPTPβ has a significant impact on subsequent signalling or protein traffic [66]. However, it was reported that mice deficient in RPTPβ show a remarkable resistance to gastric ulcer induction by VacA [67], suggesting that at least RPTPβ may play a substantial role in VacA toxicity.

### Leukocytes

Binding of VacA to T-cells and subsequent endocytosis were reported to be mediated by the integrin β_2 _subunit CD18 [36]. Together with the integrin α subunit CD11a, CD18 forms a heterodimeric transmembrane receptor on T cells named LFA-1 (lymphocyte function-associated antigen-1). Interestingly, the same protein was also reported to bind the leukotoxin of the dental pathogen *Actinobacillus actinomycetemcomitans *[68] and the *Bordetella pertussis *adenylate cyclase toxin (CyaA) [69]. LFA-1 is also exposed on the surface of other leukocytes, including granulocytes, macrophages, dendritic cells, B cells and NK cells, suggesting that the affinity for LFA-1 may be of general importance in the immun-suppressive effects of VacA [36]. However, also the functional consequences of VacA binding to LFA-1 are a matter of debate. It was argued that it is not sufficiently clear if LFA-1 primarily acts as a mediator of VacA endocytosis, or if LFA-1 is merely a mediator of intracellular signalling pathways [8]. Interestingly, new data have recently shown that uptake of VacA by T cells requires phosphorylation of the cytoplasmic tail of CD18, catalyzed by members of the protein kinase C (PKC) family [37]. There is evidence that also RPTPα may be phosphorylated by PKCs, indicating the possibility of a common mechanism of VacA internalization both in leukocytes and in epithelial cells [37].

**Figure 1:**
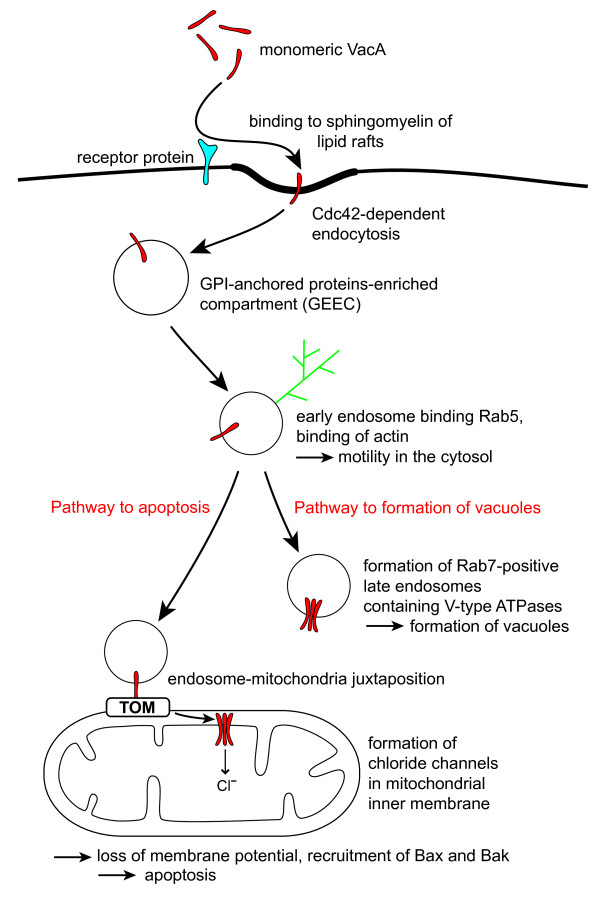
**Traffic of VacA**. Uptake of VacA into host cells is initiated by binding to lipid rafts. This step can be facilitated by receptor proteins (such as the receptor-like protein tyrosine phosphatases RPTPα and RPTPβ, or - on the surface of leukocytes - by the integrin β_2 _subunit CD18). Inside the lipid rafts, VacA binds to sphingomyelin. Subsequent endocytosis is dependent on Cdc42, a small GTPase of the Rho subfamily. The endocytosis is independent of clathrin. VacA accumulates in an early endosomal compartment enriched in GPI-anchored proteins (GEEC). The endosomes recruit cytosolic actin which forms comet-like structures. Growth of the actin fibers causes a significant motility of the endosomes in the cell. Fractions of the endosomes bind the small GTPase Rab5 and are therefore classified as early endosomes. The early endosomes undergo maturation to late endosomes, releasing Rab5 and binding Rab7. VacA in cooperation with V-type ATPases causes swelling of the endosomes and thereby a formation of vacuoles. In parallel, a fraction of the early endosomes attaches to the surface of mitochondria and VacA is transferred. VacA eventually accumulates in the mitochondrial inner membrane, forming chloride channels. Dissipation of the mitochondrial membrane potential causes recruitment of Bax and Bak, release of cytochrome c, and apoptosis. Abbreviations: Bak, Bcl-2-homologous antagonist/killer; Bax, Bcl-2-associated × protein; CD, "cluster of differentiation" (defined molecules of the cell surface); Cdc, "cell division cycle" (indicating a protein involved in the control of the cell cycle); GPI, glycosylphosphatidylinositol (a glycolipid, often attached to the C-terminus of a protein to mediate association with membranes); Rab, "Ras-related in brain" (the Rab family is part of the Ras superfamily of small GTPases); Ras, "rat sarcoma"; Rho, "ras homologous"; TOM, translocase of the mitochondrial outer membrane (a protein complex in the mitochondrial outer membrane mediating protein translocation); VacA, vacuolating cytotoxin A.

Although both LFA-1 and the phosphatases RPTPα and RPTPβ are components of signal transduction systems, there seems to be no direct participation in a signalling pathway leading to VacA-dependent apoptosis. However, binding to receptor proteins may strongly facilitate the endocytosis of VacA (Figure 1).

## The traffic route: (II) Lipid rafts and endocytosis

It has been known for a long time that after binding to a cell surface, VacA can efficiently be internalized [70]. The toxin is subsequently found in endosomes, the process is clathrin-independent [71]. In spite of uncertainties regarding the precise role of cellular receptor proteins, there is a general consensus with respect to lipid rafts as major site for uptake of VacA [8,60,65,72,73]. Sphingomyelin, a key component of the lipid rafts, was demonstrated to bind VacA directly and to facilitate its internalization [66]. VacA also binds to several glycosphingolipids, at least *in vitro *[74], but the relevance of this affinity for VacA toxicity has not been clarified.

Already in 2000 it was found that uptake of VacA depends on the presence of GPI-anchored proteins (glycosylphosphatidylinositol-anchored proteins) in the membrane [71]. GPI-anchored proteins can be delivered to a special subset of endosomes via a distinct Cdc42-dependent, clathrin-independent pinocytic pathway [75]. VacA was shown to follow this pathway and to accumulate in the corresponding GPI-anchored proteins-enriched early endosomal compartments (GEECs) [76-78]. Most GPI-anchored are subsequently recycled back to the plasma membrane. VacA avoids this step and accumulates in early endosomes that are characterized by their coating with the small GTPase Rab5.

Surprisingly, the VacA-containing early endosomes attract actin, and are subsequently moved inside the cytosol by F-actin comet tails [78]. The actin assembles preferentially at one side of an endosome, and the endosome is then propelled forward by the growth of the actin fibers. The principle of motility is similar to the mechanism of F-actin-dependent movements of *Listeria monocytogenes *inside the cytosol of host cells [79]. If the formation of F-actin is blocked, the mobility and all subsequent steps of toxicity are inhibited [78]. If actin-mediated mobility is permitted, VacA takes at least two different pathways: (1.) Most of VacA is subsequently found in vesicles that are labelled by the small GTPase Rab7 and thus defined as late endosomes. Accumulation in late endosomes initiates the formation of the vacuoles that were the reason to name the toxin the "vacuolating cytotoxin". (2.) In parallel, a smaller fraction of VacA can be transferred from the early endosomes to mitochondria. By interactions with mitochondria, VacA eventually has the opportunity to trigger apoptosis.

## The traffic route: (III) Vacuolation

If purified VacA is added to cells in culture medium, a high number of vacuoles emerge inside the cells [3,4]. It is conceivable that this drastic effect may significantly contribute to the toxicity of VacA in the infected mucosa, but surprisingly little is known about the impact of the vacuolation *in vivo*. The vacuoles resemble late endosomes, in particular they are covered with the small GTP-binding protein Rab7 and they contain V-type ATPases [80-83]. VacA was shown to primarily mediate an influx of chloride ions into the endosomes. The accumulation of negatively charged chloride ions inside the endosomes strongly facilitates the activity of the V-type ATPases. VacA thereby indirectly increases the ATP-dependent acidification of the endosomes. Due to the strong acidification, weak bases are easily trapped inside the endosomes, eventually causing an osmotic swelling of the organelles [83].

There are some data indicating that VacA may also act as a mediator of pyruvate transport [83]. However, the conductivity of the VacA ion channel is extremely low, suggesting that the inner diameter of the channel should be too small to accommodate complex molecules such as pyruvate or other organic acids [58]. Perhaps the discrepancies will be resolved once the precise structure of the VacA complexes has been elucidated. The possible permeability of VacA for pyruvate is also of relevance with respect to the question if VacA in the plasma membrane is able to mediating a release of nutrients into the mucus layer. Eventually, the permeability of VacA also determines the possible activities of VacA in mitochondria.

## The traffic route: (IV) Mitochondria

For a long time, intracellular VacA was only detected in endosomes and in vacuoles. The first attempts to express VacA in the cytosol of HeLa cells confirmed this concept. VacA was found to co-localize with the V-ATPases in the membranes of vacuoles [84]. A few years later, the group of Patrice Boquet decided to express different fusion proteins of VacA containing a green fluorescent protein (GFP) domain in HeLa cells and to determine their distribution. Surprisingly, the experiments indicated that VacA or parts of VacA may be transported to mitochondria [5]. It was found that the C-terminal part of VacA (named p55 or p58) was retained in the cytosol. However, the N-terminal part of VacA (named p34 or p37) localized to mitochondria, at least if a GFP moiety was attached to the N-terminus. Moreover, the experiments revealed that constructs of p34 carrying the GFP moiety at the C-terminus - and thus exposing a free N-terminus - triggered apoptosis of the cells. Apoptosis was initiated by release of cytochrome c, it could be inhibited by Bcl-2 [5].

However, in these experiments, the VacA constructs were only shown to target mitochondria if synthesized in the cytosol. Unfortunately, it was completely unclear how VacA may ever encounter mitochondria if provided by bacteria, or if contained in endosomes. Independent studies then confirmed that purified VacA was an efficient mediator of apoptosis if added to intact cells from the outside [6,24], and a series of carefully conducted studies demonstrated that externally added VacA was indeed transported to mitochondria, causing dissipation of the mitochondrial membrane potential, release of cytochrome c and apoptosis [25,26,85].

Although it was obvious that VacA can trigger apoptosis by interactions with mitochondria, the molecular mechanisms continued to be enigmatic [4,31]. Several questions were completely unclear: What is the mitochondrial targeting signal of VacA? What is the target structure of VacA inside the mitochondria? What is the pathway of VacA from the plasma membrane to the mitochondria? What is the mechanism of cytochrome c release if VacA enters mitochondria? These questions were recently addressed by several new studies.

### What is the mitochondrial targeting signal of VacA?

The biogenesis of endogenous mitochondrial proteins is very well characterized [86,87]. Several different mitochondrial targeting signals have been identified. These can be located at the N-terminus or at the C-terminus of a protein, and also internal targeting signals are known [87]. No such signal sequence is contained in the primary structure of VacA. By testing different segments of VacA for mitochondrial targeting in intact cells and in a cell-free system *in vitro*, it turned out that the 32 amino acid residues of the VacA N-terminus are necessary and sufficient for targeting to mitochondria [58].

This part of VacA represents a new and peculiar mitochondrial targeting sequence, there are no obvious similarities to any endogenous mitochondrial proteins. The mitochondrial targeting sequence of VacA does not contain any charged residues, most residues are hydrophobic. The sequence targets VacA to the TOM complex of the mitochondrial outer membrane (the translocase of the mitochondrial outer membrane) which mediates the import of mitochondrial precursor proteins from the cytosol. The VacA N-terminus had previously been known to be essential in the formation of vacuoles [3,4], and it had been suggested that the N-terminus may form the ion channel in VacA oligomers [88]. However, the more recent data indicate that pore-formation by VacA is independent of the N-terminus. It was found that the N-terminal domain of VacA (p34, residues 1-311) is able to form the characteristic anion channel of low conductivity even if the N-terminal residues 1-32 are completely deleted [58]. It is therefore conceivable that the hydrophobic VacA N-terminus primarily serves as a targeting signal and as a mediator of membrane insertion. In fact, there is some experimental evidence that the VacA N-terminus is also in vacuoles primarily required for efficient membrane insertion and not for oligomerization or pore formation [89].

### What is the target structure of VacA inside the mitochondria?

The hydrophobic N-terminus of the VacA toxin is not simply a mediator of unspecific insertion into the mitochondrial outer membrane. Two independent studies found that VacA seems to exclusively accumulate in the mitochondrial inner membrane [58,90]. The mechanism that directs VacA specifically to the inner membrane is still unknown. However, the localization has important implications. Import of VacA into mitochondria quickly entails a dissipation of the mitochondrial membrane potential [5,25]. This effect is probably caused by the conductivity of the VacA ion channel in the inner membrane. In a first step, chloride ions should diffuse into the mitochondrial matrix, causing a hyperpolarization of the membrane potential. In a second step, the function of the mitochondrial respiratory chain appears to be compromised, causing an irreversible loss of the membrane potential.

### What is the pathway of VacA from the plasma membrane to the mitochondria?

For 10 years it had been difficult to reconcile the actions of VacA on mitochondria with the obvious tendency of externally added VacA to accumulate in endosomes. The study of Gauthier et al. (ref. [78]) demonstrated a striking mobility of the endosomes in the cytosol, raising the question if VacA may be transferred from the endosomes to mitochondria upon direct contact. Experimental evidence supporting this possibility was provided only recently by a highly significant study [91]. The authors investigated the pathway of externally added VacA from the plasma membrane into the interior of mouse embryonic fibroblasts and determined the intracellular location of VacA at different time points. In these experiments, VacA-containing endosomes were observed in direct contact with mitochondria. The endosome-mitochondria juxtaposition was strictly dependent on VacA, but surprisingly also on a recruitment of Bax and Bak to the endosomal membranes. After about 12 hours, significant amounts of VacA were detected in the mitochondrial fraction, clearly preceding the induction of apoptosis. The transfer to mitochondria was completely blocked with mutant versions of VacA containing an amino acid exchange within the N-terminal 32 residues (P9A or G14A, respectively). Based on previous assumptions, the authors proposed that the transfer might be dependent on the VacA channel activity. However, taking into account the notion of the VacA N-terminus acting as a targeting signal [58], it is more likely that the N-terminus is involved in direct interactions with the mitochondrial protein import machinery [7]. Eventually, it is also remarkable that the authors showed a transfer of the entire holo-toxin to the mitochondria. In previous studies it had been unclear if the complete VacA, or possibly only the toxic N-terminal domain enters the mitochondria.

## The mechanism of cytochrome c release: VacA and apoptosis

Why is apoptosis a consequence of VacA import into mitochondria? As discussed in this review, there is now direct evidence of VacA targeting the mitochondrial inner membrane, and ample evidence of VacA forming anion channels. An enzymatic activity of VacA seems to be lacking, and there is no indication that VacA may act by direct interactions with cellular enzymes or protein complexes that are directly involved in the regulation of the cell cycle. Hence, the formation of an anion channel of low conductivity in the mitochondrial inner membrane is currently the only activity of VacA in mitochondria that is supported by the available data. If this is true, VacA-dependent apoptosis should efficiently be blocked by reagents that block the VacA channel activity. Interestingly, experiments of this type have already been conducted. NPPB (5-nitro-2-[3-phenylpropylamino] benzoic acid) is a chloride channel blocker that completely inhibits the conductivity of VacA [58,92,93]. If cells are pre-incubated with this reagent, they are resistant against VacA-dependent apoptosis [25]. This correlation is currently the best evidence of an essential role of the VacA ion channel in VacA-dependent apoptosis.

Strikingly, *H. pylori *itself is able to actively suppress the apoptotic action of its VacA toxin. Cells that receive the *H. pylori *CagA protein (by type IV-secretion; see the contribution of S. Backert in this issue) are resistant against VacA. CagA initiates a signalling cascade that stops the uptake of VacA in an early step, probably in the GPI-anchored proteins-enriched early endosomal compartments [94]. The effect of CagA confirms the relevance of an unrestricted access of VacA to mitochondria in VacA-induced apoptosis.

Concerning the mechanistic relation of ion channel formation in the mitochondrial inner membrane to subsequent apoptosis, essentially three points have been clarified: (i) Import of VacA causes loss of the mitochondrial membrane potential [5,25,85], (ii) members of the Bcl-2 family, including Bax and Bak are intrinsically involved in VacA-dependent apoptosis [5,26,29,30,91,95], and (iii) cytochrome c and other mitochondrial proteins are released into the cytosol [5,26,29,31,85].

Unfortunately, data are lacking to explain why chloride influx into the mitochondrial matrix causes Bax and Bak recruitment at the mitochondrial outer surface. The functional relation of these two events is currently the missing link in research on VacA-induced apoptosis. It is known that any dissipation of the mitochondrial membrane potential can be sufficient to cause dramatic structural changes within the mitochondria, including a significant redistribution of pro-apoptotic factors [96-98]. However, it seems to be difficult to explain these consequences in molecular terms. The difficulties are in part due to the fact that in general only very little is known about the communication between the mitochondrial inner and outer membrane. Further research on the functions of the VacA toxin is required to elucidate this relation.

## Conclusions

The epithelial cells of the gastric mucosa undergo apoptosis after a few days irrespective of any infection. In the beginning it was therefore difficult to determine if *H. pylori *interferes with this process by specific means or only as an unspecific mediator of inflammation. It is now well established that *H. pylori *can trigger apoptosis both by the death receptor pathway and by the mitochondrial pathway. In recent years, the mitochondrial pathway received most attention after if had been found that the VacA toxin of *H. pylori *can efficiently trigger apoptosis by direct interactions with mitochondria. The pathway of VacA from the plasma membrane to the mitochondria of the host cells had been enigmatic for 10 years. Most data suggested that VacA should bypass the mitochondria and accumulate exclusively in late endosomes. Only very recently, in this respect several important questions could be answered (Figure 1): Following uptake at lipid rafts, VacA is contained in GPI-anchored proteins-enriched early endosomal compartments. VacA appears to stay in endosomal membranes, there is no evidence that substantial amounts of VacA are released into the cytosol. However, early endosomes containg VacA can recruit actin at their outer surface, tails of F-actin can form at one pole of the endosomes, and together with the endosomes, VacA has a remarkable motility inside the cell. VacA is transferred to mitochondria in direct contact between endosomes and mitochondria. Inside the mitochondria, VacA accumulates in the inner membrane, obviously forming an anion channel of low conductivity. Subsequent apoptosis is essentially a consequence of the formation of a chloride channel in the mitochondrial inner membrane. Transfer to the mitochondria and insertion into the mitochondrial inner membrane is mediated by the VacA N-terminus. Apotosis is initiated by dissipation of the mitochondrial membrane potential, recruitment of Bax and Bak, and release of cytochrome c and other pro-apoptotic proteins into the cytosol.

The next three big questions to be answered are probably the following: (1.) What exactly is the structure and the topology of the VacA complexes in the membranes? (2.) What is the mechanism of the transfer of VacA from the endosomes to the mitochondria? (3.) What is the mechanism that links the formation of the ion channel in the mitochondrial inner membrane to the activation of Bax and Bak and to the release of pro-apoptotic factors? Perhaps it will again take 10 years to answer these questions. However, answers to these questions would not only clarify a central mechanism of *H. pylori*-induced gastro-duodenal diseases, they should also help to understand the mechanisms of apoptosis in general.

## Competing interests

The author declares that they have no competing interests.
